# What’s in My Coffee? Do-It-Yourself Testing for Chicory Adulteration Using Particle Trapping in Stencil-Based Paper Devices

**DOI:** 10.1021/acsomega.5c04540

**Published:** 2025-07-09

**Authors:** Balachandar Sundarrajan, Priyadharshini Shanmugam, Anusha Prabhu, Thangaraju Dheivasigamani, Naresh Kumar Mani

**Affiliations:** † Microfluidics, Sensors and Diagnostics (μSenD) Laboratory, Centre for Microfluidics, Biomarkers, Photoceutics and Sensors (μBioPS), Department of Biotechnology, 76793 Manipal Institute of Technology, Manipal Academy of Higher Education, Manipal, Karnataka 576104, India; ‡ Nanocrystal Design and Application Lab (n-DAL), Department of Physics, 471340 PSG Institute of Technology and Applied Research, Coimbatore, Tamil Nadu 641062, India; § Centre for Advanced Materials, PSG College of Arts & Science, Coimbatore, Tamil Nadu 641014, India

## Abstract

Our study introduces a cost-effective Do-It-Yourself (DIY) method for detecting chicory adulteration in coffee powder via the Shake & Invert approach integrated with a particle trapping mechanism. Coffee powders of different sizes were blended with chicory in distinct proportions. A bioline layer was employed as an adhering agent to selectively capture chicory on a laminated paper-based device. This selective adhesion is attributed primarily to the differences in surface morphology between the coffee and chicory particles. The texture and structural arrangement of the particles play crucial roles in determining their interaction with the bioline-coated surface. SEM imaging provided further insight into these differences, revealing that chicory possesses rough and distinctive fiber-like structures, whereas the coffee particles have a uniform plate-like surface. These structural characteristics are believed to enhance its adhesion toward the bioline-coated paper, resulting in superior adhesion compared with that of the coffee particles. Our developed approach exhibited a limit of detection (LOD) at a coffee-to-chicory ratio of 90:10. This understanding of the distinct physical properties of chicory and coffee underscores the effectiveness of our adhesive testing method, indicating its potential as a robust tool for detecting chicory within 5 min. This “low-cost” sensing could facilitate accessible screening of coffee samples, enhancing quality within the industry and ultimately safeguarding consumer interests.

## Graphical abstract

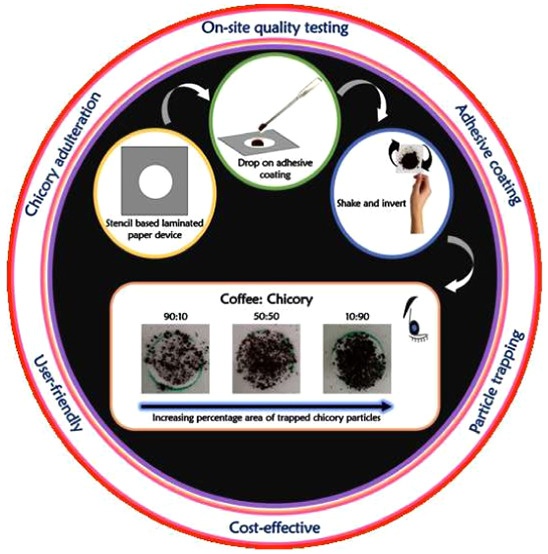

## 1 Introduction

Coffee is among the most widely consumed beverages in the world, particularly in nontraditional markets;^1^ it is known for its attractive aroma and flavor and for refreshing and invigorating the feeling it provides to the consumer.^2^ Additionally, studies have reported numerous benefits of coffee, including its antioxidant properties, the presence of chlorogenic acids and the role of its degradation products in cardiovascular illnesses,^3^ as well as the deceleration of the progression of neurodegenerative disorders such as Parkinson’s disease.^2^ Furthermore, coffee has been found to play a role in enhancing the fecal microbiota and promoting effective fat excretion in feces.^4^ It also acts as a chemoprotective agent, impeding various stages in the development of cancers. However, owing to the wide range of varieties of crops available on the market, the striking differences that exist between their prices, and the noticeable increase in coffee prices,^5^ there is a considerable risk of both inadvertent and deliberate adulteration of coffee. Hence, the beneficial aspects of coffee are subject to the purity and quality of the coffee being consumed, making it essential to have a quality check of the coffee powders sold on the commercial market.

The adulteration of coffee is performed either with inferior species of coffee varieties^6^ or with noncoffee additives.^7^ More than 100 products are being used as adulterants in coffee, with corn, rice, wheat, soybean, barley and chicory being used extensively, owing to their lower cost and structural similarity with ground or roasted coffee in their respective forms.^8,9^ Chicory (*Chicorium intybus* var. *sativum*) root powder, which strongly contributes to the flavor enhancement of coffee, is added as an adulterant in countries such as the USA, Brazil, France and India. Consequently, this enables deceitful economic profits in exchange for adulterated coffee products.^10^ Despite these economic benefits, chicory powder contains a high acrylamide content, which can lead to adverse health effects when consumed on a daily basis, in addition to being obviously illegitimate in nature and posing a serious threat to future economic advancements in the coffee industry in general.^11–14^ Additionally, the presence of chicory can cause severe consequences due to allergic reactions, stimulate bile fluid production in people with gallstones and cause gallbladder cancer. It is considered unsafe for pregnant women.^15^ Thus, the only prominent solution to minimize this food fraud is to develop robust and highly efficient analytical methods^16–20^ designed to detect the presence of the adulterant chicory in coffee, with the aim of subsequently circumventing the problems associated with economic losses and uncertainty in the food industry overall.

There are a plethora of successfully validated scientific techniques for the sensitive and accurate detection of adulterants such as chicory in coffee products, which rely on either the presence of specific groups of chemicals of the same family (targeted methods) or the assessment of instrumental responses from the samples without any previous knowledge about the chemical composition (untargeted or fingerprinting methods).^21^ Some of these methodologies include microscopy, infrared spectroscopy, multispectral imaging, mass spectrometry and nuclear magnetic resonance (NMR).^7,10^ Chromatographic techniques, particularly high-performance liquid chromatography (HPLC), gas chromatography (GC), and capillary electrophoresis (CE), are among the most extensively utilized methods.^10,13^ Sensitive and efficient, the above-mentioned approaches still suffer from the limitations of requiring expensive equipment and operation, expertise in interpreting the results and manpower,^22,23^ a large number of characterized samples,^5^ and multiple intermediate steps,^6^ thereby restricting their applicability within the boundaries of the laboratory. In addition, tests involving larger numbers of samples belonging to different producers are needed to validate the performance of methods such as HPLC.

This underscores the necessity for the development of a simple, single-step, cost-effective approach for the sensitive and convenient detection of chicory adulteration in coffee. In this study, for the first time, we demonstrated that cost-effective Do-It-Yourself (DIY) adhesive testing can be aimed at detecting chicory adulteration in coffee samples. A schematic illustration showing the “Shake and Invert” technique for detecting chicory adulteration in coffee powder is given in Figure 1.

**Figure 1:**
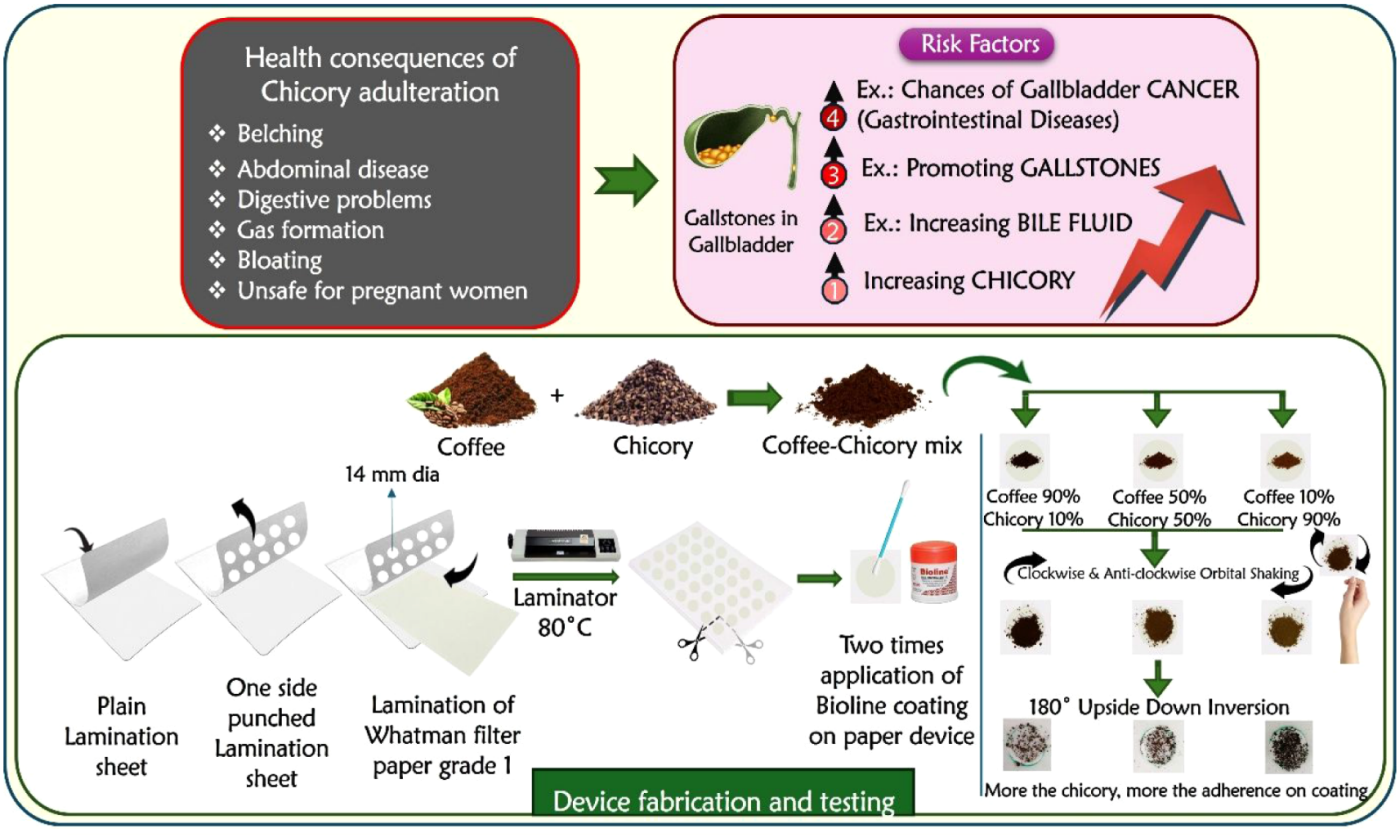
Schematic illustration showing laminated paper-based device for detecting chicory adulteration in coffee powder via the SHAKE and INVERT approach.

## 2 Materials and Methods

Coffee powder of various sizes was purchased from a local vendor and was named C1, C6 and C8 in increasing order of size such that C1 represents finely ground coffee powder, whereas C8 represents roughly ground coffee powder. Chicory (Ch) was procured from the local market of Coimbatore, southern India. Bioline (http://www.biopharmgroup.co.in/product/bioline-100g/) was procured from a local pharmacy, Manipal, India. Whatman filter paper (Grade 1) was purchased from Whatman, India. Lamination sheets were purchased from a local stationery shop in Mangaluru, India.

### 2.1 Preparation of the Coffee Chicory Samples

The three different coffee samples (C1, C6, and C8) were mixed individually with chicory (Ch) at ratios of 90:10, 50:50 and 10:90. The coffee and chicory powders were weighed separately at the above ratios (0.18 g: 0.02 g, 0.1 g: 0.1 g, 0.02 g: 0.18 g, respectively) and mixed properly via a spatula.

### 2.2 Characterization of Chicory and Coffee Powder

X-ray diffraction (XRD) analysis was conducted to elucidate the structural composition of both the coffee and chicory samples. Small quantities of coffee and chicory were carefully placed onto clean sample holders, ensuring an even distribution across their surfaces. The analysis was carried out via an Empyrean X-ray diffractometer (Malvern PANalytical) utilizing CuKα radiation with a wavelength of λ = 1.54 Å. After precise alignment of the sample holder, the XRD scan was initiated, and diffraction patterns were recorded for both samples. Peaks corresponding to distinct compounds were discerned based on their 2θ values. Subsequent examination of peak intensity and peak width provided valuable insights into the crystalline and amorphous characteristics of the samples.

The surface morphology and structure of both chicory and coffee powders were examined through SEM analysis via a ZEISS Sigma Field Emission Scanning Electron Microscope (FESEM). The investigation included various magnifications (500×, 1k×, 2.5k×, 5k×, and 10k×), with multiple images captured to ensure comprehensive and representative results. The acquired microscopy images were subsequently stored for analysis and interpretation.

### 2.3 Development of Bioline-Coated Paper-Based Devices for Chicory Testing

Circles with a diameter of 14 mm were marked on the upper layer of the lamination sheet via a Pro-Circle, and the marked regions were punched to obtain holes. A Whatman filter paper sheet (A4 size) was placed between the two layers of the lamination sheet, and hot lamination was performed via a Kent Laminator at 80 °C. The sheet was then cut into individual paper-based devices with exposed circular zones. A cotton swab was used to apply bioline (the main component is white petroleum jelly) two times over the circular area of the exposed paper zones and further used for chicory testing.

### 2.4 Detection of the Chicory Content in Coffee via Paper-Based Devices

One micro spatula of the prepared proportions of coffee-chicory mixtures was dropped onto the circular zones of the bioline-coated paper devices, which were shaken in orbital motion and inverted 180° to drop the unbound particles. Triplicate trials of the experiment were performed for each sample to ensure reproducibility. Images depicting the particle trapping of chicory present in coffee at different ratios onto bioline-coated devices were captured appropriately via a smartphone. To quantify the area coverage of particles adhered to the bioline-coated circular paper zones, the captured images were first cropped into rectangular shapes of the same size, covering the area of trapped particles. Next, the cropped images were analyzed via FIJI software to determine the percentage area of the adhered particles.

### 2.5 Testing with Commercial Coffee Samples

Different commercial coffee powders were procured from local grocery stores. One micro spatula of the powder was added onto the bioline-coated paper device, which was shaken in orbital motion and inverted to remove the unbound particles. Further images were subjected for analyzed to assess the percentage area of trapped particles using FIJI software (as per the protocol mentioned in Sections 2.4 and 2.6).

### 2.6 Image Analysis via Fiji Software

The rectangular cropped images of the trapped particles in the bioline-coated paper zones were opened in Fiji software, and the color threshold (Hue) was adjusted to include all the particles (Image → Adjust → Color Threshold). The colored images were then converted to binary images (Process → Binary → Make Binary) and inverted (Edit → Invert). The percentage area of all the trapped/adhered particles was checked by analyzing the inverted binary images (Analyze → Set Measurements → Check Area fraction → Ok and Analyze → Measure).

## 3 Results and Discussion

### 3.1 Powder X-ray Diffraction Analysis

X-ray diffraction (XRD) analysis of both the coffee and chicory samples (Figure 2) provided significant insights into their respective structural characteristics. The XRD patterns are depicted in Figure 2. revealed the semicrystalline nature of both the coffee and chicory samples. This is evident from the presence of well-defined sharp peaks, indicating crystalline structures, and broad peaks indicative of amorphous attributes.^24,25^ Specifically, the XRD profile of the coffee sample displays a prominent sharp peak at a diffraction angle of 11.91° coupled with a broad peak at 20.46°. These features align consistently with those typically associated with roasted coffee, underscoring the influence of thermal treatment on the crystalline nature of coffee particles. Conversely, the XRD pattern of chicory presents distinctive peaks at 17.55°, 21.22°, 26.65°, and 31.40°. The sharp peak at 26.65° may be attributed to the presence of carbon, a byproduct of the roasting process applied to chicory.

**Figure 2:**
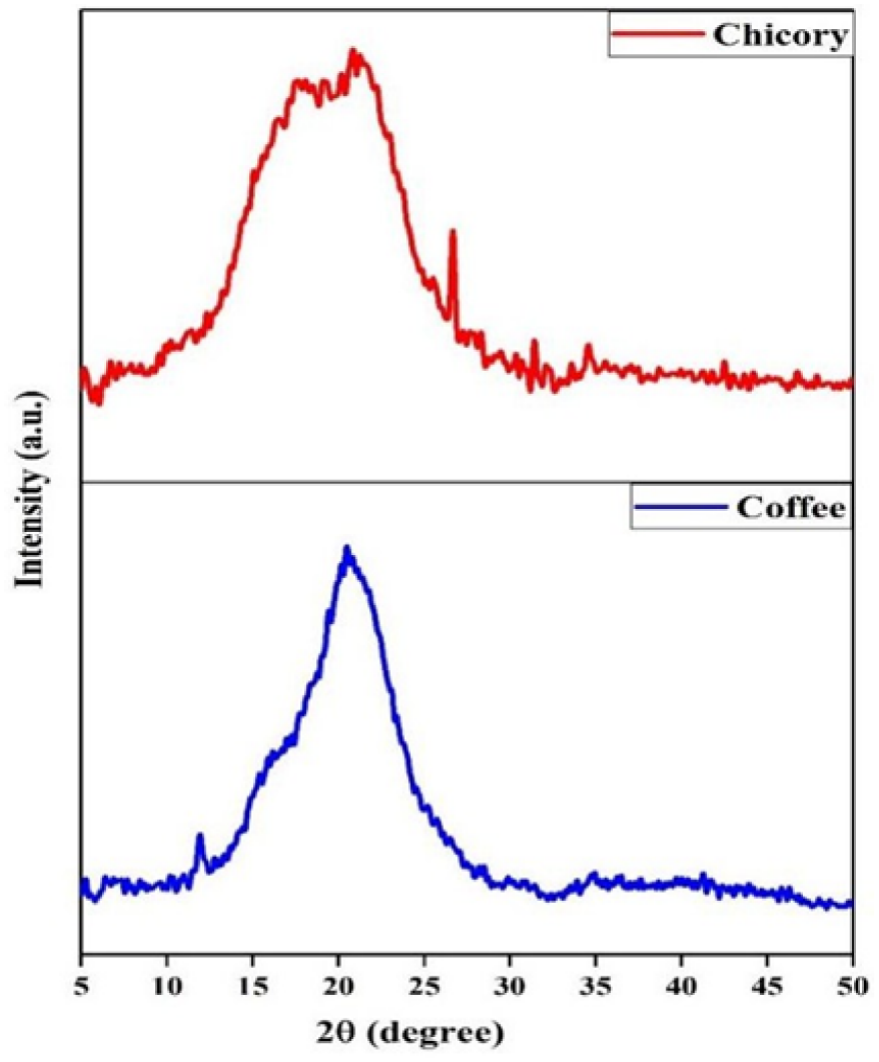
Comparative XRD pattern of chicory and coffee.

### 3.2 Surface Morphology Analysis

SEM analysis revealed the surface morphological attributes of the coffee and chicory samples. The SEM images of C1, which is a finely ground coffee powder, as depicted in Figure 3C1­(a–c), reveal finer smooth and uniform surface particles. Moreover, the surface morphology displayed a flaky texture, possibly indicative of underlying chemical transformations during the roasting process, culminating in the creation of a porous framework. In stark contrast, C8, characterized by its coarse texture, exhibited a distinctly cracked and rigid composition (Figure 3d–f), featuring unevenness in comparison to C1. Figure 3Ch­(g–i) shows the surface morphology of chicory, which has a continuous and irregular surface texture and agglomerated particles. From the SEM images, the particle size distributions of the samples were estimated and are depicted in Figure 3a,d,g. The average particle size of the C1 sample is 37.48 μm, whereas that of C8 is 121.55 μm. Furthermore, the chicory particles have an average size of 88.44 μm. Although chicory particles are slightly larger than finely ground coffee C1 particles, their rough surface texture might result in more adhesion to the bioline layer. Thus, the different surface morphologies and particle sizes of the chicory and coffee samples may influence the adhesive behavior of the samples with a bioline layer.

**Figure 3:**
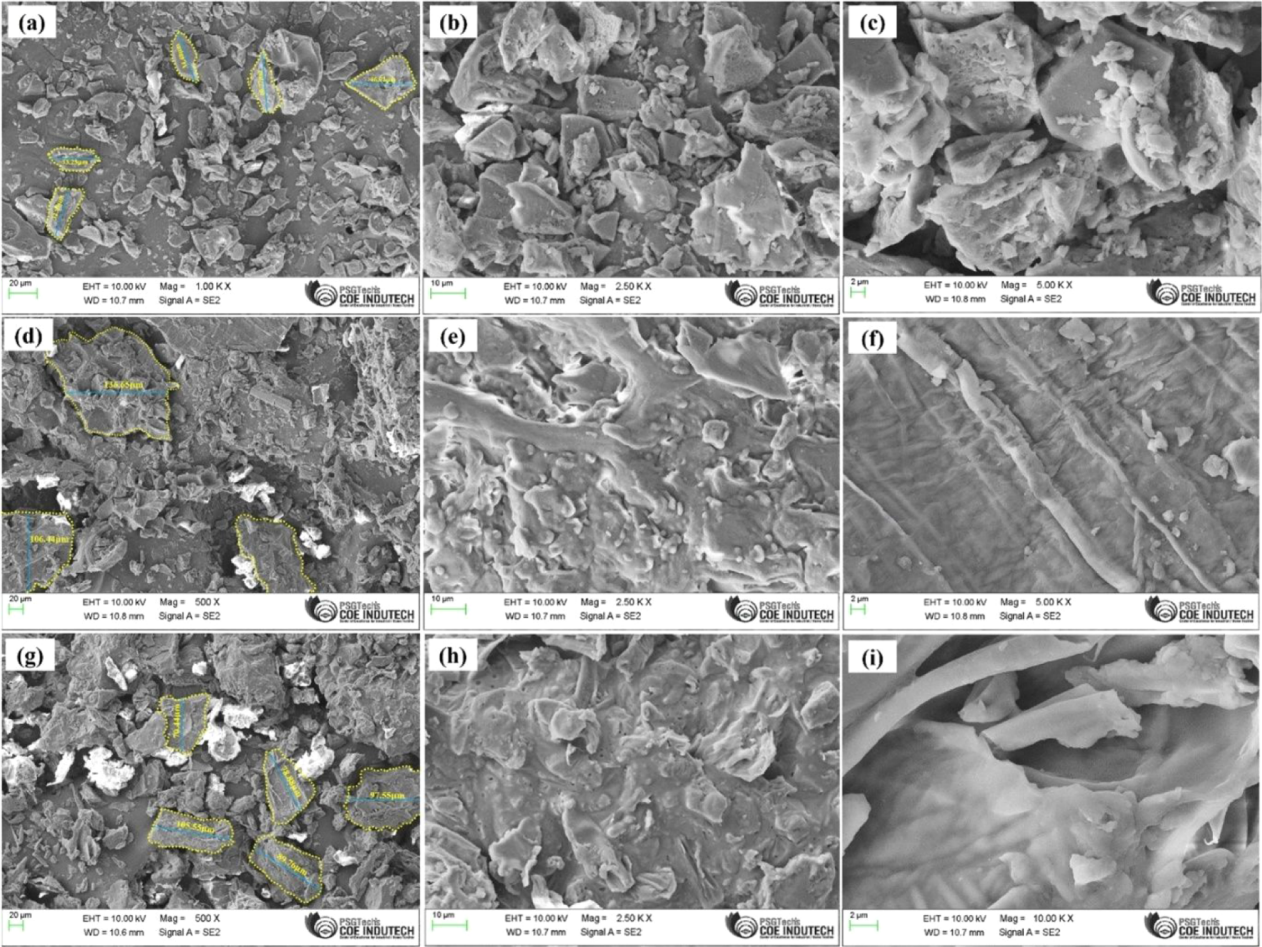
SEM images of coffee (C1­(a–c), C8­(d–f)) and chicory (Ch­(g–i).

### 3.3 Detection of Chicory Content in Coffee via Bioline-Coated Paper-Based Devices

Different coffee powders (C1, C6 and C8) with varying grind sizes were acquired and blended with chicory (Ch) in different proportions. Subsequently, we employed bioline as an adhering agent by coating it onto circular zones on a laminated paper-based device. Following the application of the prepared coffee-chicory mixed samples onto paper devices, they were moved in orbital motion and gently inverted to drop the unbound particles. The results presented in Table 1 offer a visual depiction of particle trapping on a bioline-coated paper device, showing the interaction between three different coffee powders (C1, C6 and C8) blended with Ch at different ratios, specifically the 90:10, 50:50 and 10:90 ratios. Notably, pure chicory (Ch) exhibited the highest level of adherence to the bioline layer, surpassing all the other samples. Conversely, across all types of coffee powders, the ratios of coffee to chicory (90:10, 50:50 and 10:90) consistently demonstrated differing levels of particle trapping. Among these ratios, the 10:90 ratio consistently demonstrated the highest level of adherence, followed by the 50:50 and 90:10 ratios. In contrast, pure coffee consistently exhibited a lower degree of adherence to chicory content than did 50:50 coffee. The observed difference in adherence between coffee and chicory particles can be attributed to distinct variations in their surface morphology. Chicory has a continuous, irregular, and fibrous structure that enhances its contact with the bioline layer, thereby promoting stronger physical entrapment and more effective physical anchoring. Thus, the morphological features of chicory enable more effective interactions with the bioline layer, resulting in superior adhesion. In contrast, coffee particles possess a uniform plate-like surface, which limits physical interaction with the bioline layer. This reduced contact with the bioline layer led to weaker adhesion and a lower trapping efficiency of the coffee particles. Hence, chicory has greater trapping efficiency than does coffee, even when both are exposed to the same bioline-coated surface under identical conditions.

**Table 1:**
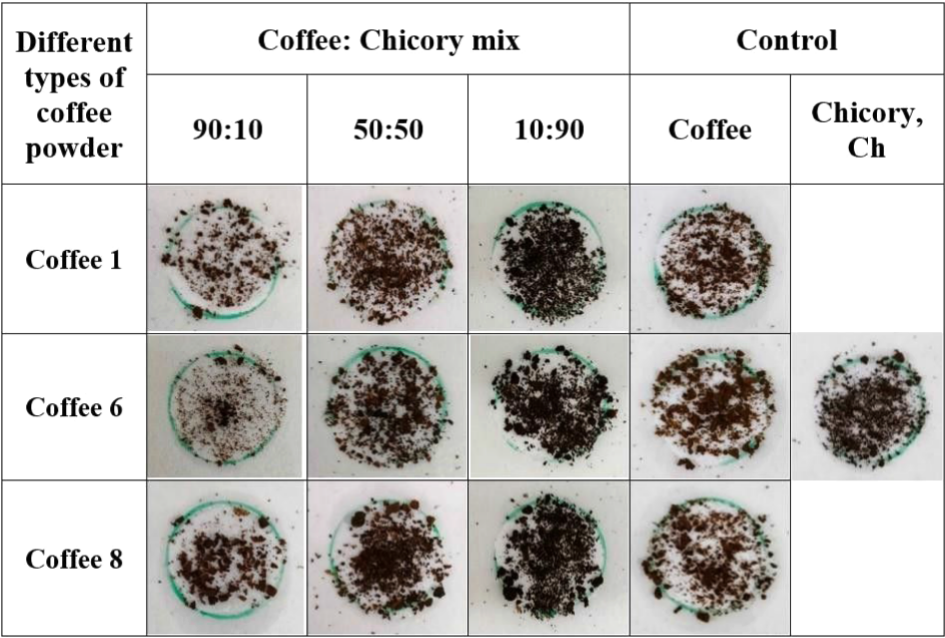
Adherence Behaviour of Coffee and Chicory Samples on Bioline-Coated Paper-Based Device, Three Distinct Coffee Powders (C1, C6, and C8) Were Combined in Different Proportions

The percentage area data obtained from the image processing of trapped particles (C1: Ch, C6: Ch, C8: Ch) in paper devices through FIJI software are graphically represented in Figure 4. Notably, across all three coffee samples, the 10:90 coffee-to-chicory ratio resulted in the highest degree of particle trapping on the bioline-coated surface, followed by the 50:50 and 90:10 ratios. The observed adherence trend remained invariant, even when confronted with variations in coffee particle sizes. Concurrently, a discernible reduction in the quantity of adhered particles was noted with decreasing chicory content, providing a clear indication that chicory powder adheres more effectively to the bioline layer than does coffee powder. As previously outlined, the chemical characteristics and surface properties of coffee and chicory influence their adherence to the bioline layer. Furthermore, we performed experiments with two (∼0.06 g) or three (∼0.09 g) samples to confirm the area of adherence of the chicory particles. There is a variation in the adherence based on the sample quantity; hence, the quantity of the sample used for testing should be maintained at ∼0.03 g (1 micro spatula). The adherence of chicory obtained with different quantities of coffee samples 1 and 6 added to the device is shown in Figure S1.

**Figure 4:**
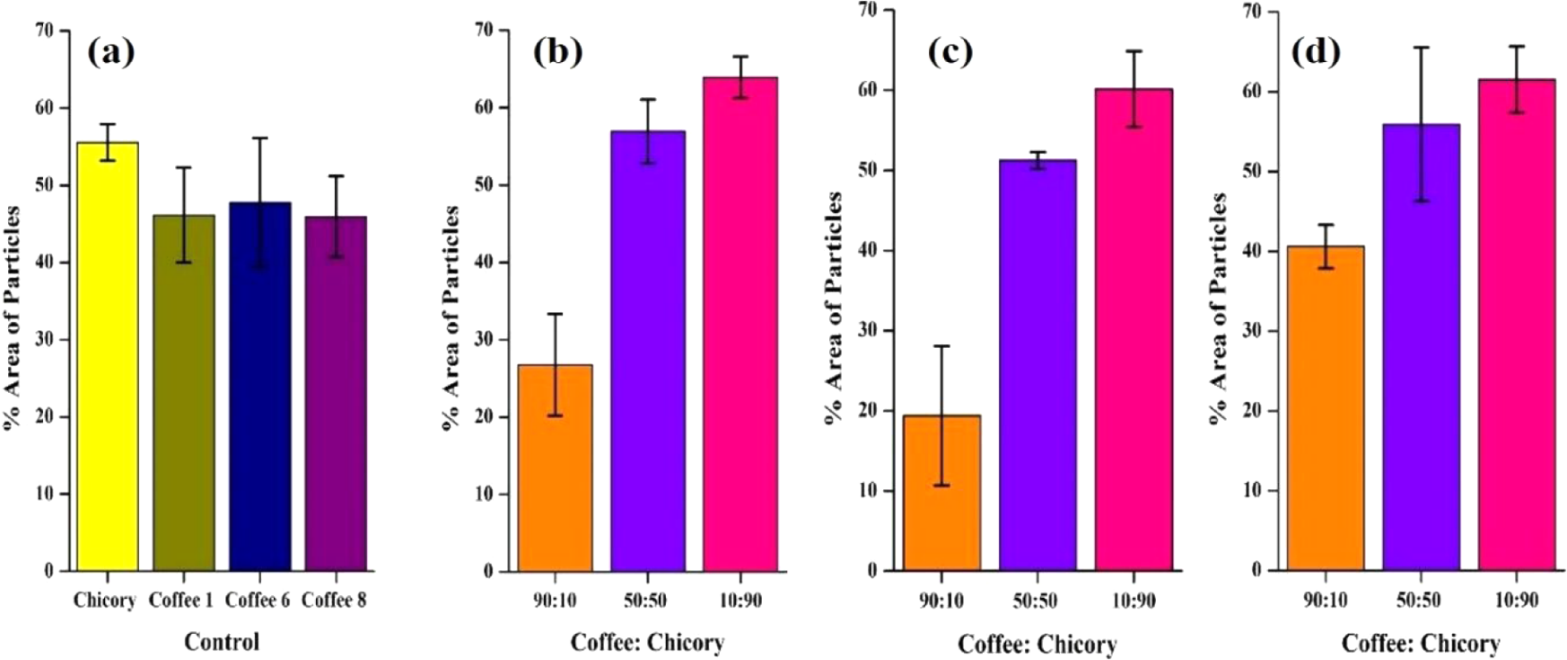
Percentage area adherence of coffee and chicory particles in paper-based devices (a) Control b) C1:Ch (c) C6:Ch (d) C8:Ch.

From the bar graphs (Figure 4), it can be inferred that chicory particles exhibit a greater affinity for the bioline layer than their coffee counterparts in the coffee-chicory mixture do. In contrast, pure coffee without chicory showed less adherence than did 50:50 coffee to the chicory mixture. As per the FSSAI guidelines, the chicory content of coffee should not exceed the permissible limit of 49% (File No. RCD-15001/6/2021-Regulatory- FSSAI-Part(1)). Hence, the particle trapping of pure coffee particles in bioline-coated paper devices with a chicory content of less than 50:50 corroborates this limit, aiding in the identification of chicory adulteration in more than 49% of the coffee powder. Additionally, the consistent trapping pattern of the chicory samples mixed with various sizes of coffee particles indicates uniform behavior in terms of their adhesion properties regardless of their individual sizes. In conclusion, this Do-It-Yourself method of visualizing and quantifying particle adhesion serves as a reliable and cost-effective means for detecting chicory adulteration in coffee powders. This developed DIY paper device can contribute to the United Nations Sustainable Development Goal, which focuses on good health and well-being.^26^

### 3.4 Testing with Commercial Coffee Samples

To validate the reliability of the reported chicory adulteration detection method using bioline layer-coated paper-based devices, tests were conducted on four commercially available coffee powder samples (Figures 5 and S2). As previously stated, the Food Safety and Standards Authority of India (FSSAI) mandates that the chicory content of coffee must not exceed 49%. Analysis via the bioline layer-coated stencil-based paper device revealed that the particle adhesion area on the bioline layer remained below 45% for all four commercial coffee samples, indicating that the chicory content in each sample was less than 45%. This result not only complies with the regulatory limit but also demonstrates the ability of our method to effectively quantify chicory content. The simplicity, cost-effectiveness, and accuracy of this approach highlight its potential as a practical tool for routine screening of chicory adulteration in commercial coffee powders.

**Figure 5:**
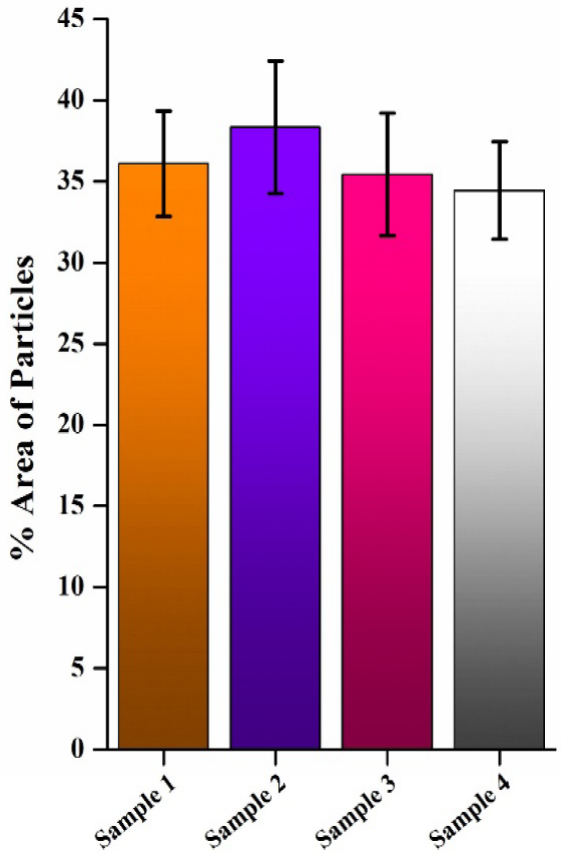
Percentage area adherence of commercial coffee powders in paper-based devices.

## 4 Conclusion

Our research introduces an accessible Do-It-Yourself (DIY) technique for detecting chicory adulteration in coffee samples in less than 5 min. Through this study, a novel particle trapping technique using an adhesive-based coating in stencil-integrated paper devices was realized, revealing significant insights into the adhesion of chicory within a bioline-coated area. This emphasizes the efficacy of the employed adherence properties, revealing the selective trapping of chicory particles in the coffee-chicory mixture onto the adhering agent. This DIY approach presents a promising tool for the rapid and cost-effective screening of coffee authenticity, which has significant implications for quality control within the coffee industry as well as for other sensing applications.^27–32^ The mitigation of chicory adulteration not only upholds product integrity but also has broader socioeconomic implications, bolstering livelihoods and nurturing a fair market environment. Moreover, ensuring the purity of coffee products on a global scale holds paramount importance for public health. In addition, exploring automated smartphone-based testing of particle trapping as a potential avenue for future research could further enhance the practical application of this method.

## Supplementary Material



## Notes

Figure S1 showing area adherence of different amounts of C1 and C6 coffee-chicory samples, and Figure S2 depicting the tests with commercial coffee samples (PDF)
