# Supplementary material for: What’s in My Coffee? Do-It-Yourself Testing for Chicory Adulteration Using Particle Trapping in Stencil-Based Paper Devices
Source: ACS Omega. 2025 Jul 9;10(28):31059–65. doi: 10.1021/acsomega.5c04540 (PMC12290975; doi:10.1021/acsomega.5c04540)
Supplement: Supplementary file 1 [file ao5c04540_si_001.pdf]

What's in my coffee? Do-It-Yourself Testing for Chicory Adulteration via Particle Trapping in Stencil-based Paper Devices

Balachandar Sundarrajan[a±], Priyadharshini Shanmugam[b,c±], Anusha Prabhu[a±], Thangaraju Dheivasigamani[b], Naresh Kumar Mani*[a]

[a.] *Microfluidics, Sensors and Diagnostics (μSenD) Laboratory, Centre for Microfluidics, Biomarkers, Photoceutics and Sensors (μBioPS), Department of Biotechnology, Manipal Institute of Technology, Manipal Academy of Higher Education, Manipal, Karnataka, 576104, India*

[b.] *Nanocrystal Design and Application Lab (n-DAL), Department of Physics, PSG Institute of Technology and Applied Research, Coimbatore-641062, Tamil Nadu, India.*

[c] *Centre for Advanced Materials, PSG College of Arts & Science, Coimbatore – 641014, Tamil Nadu, India*

± *Equally contributed.*

* Corresponding author

E-mail address: naresh.mani@manipal.edu

maninaresh@gmail.com

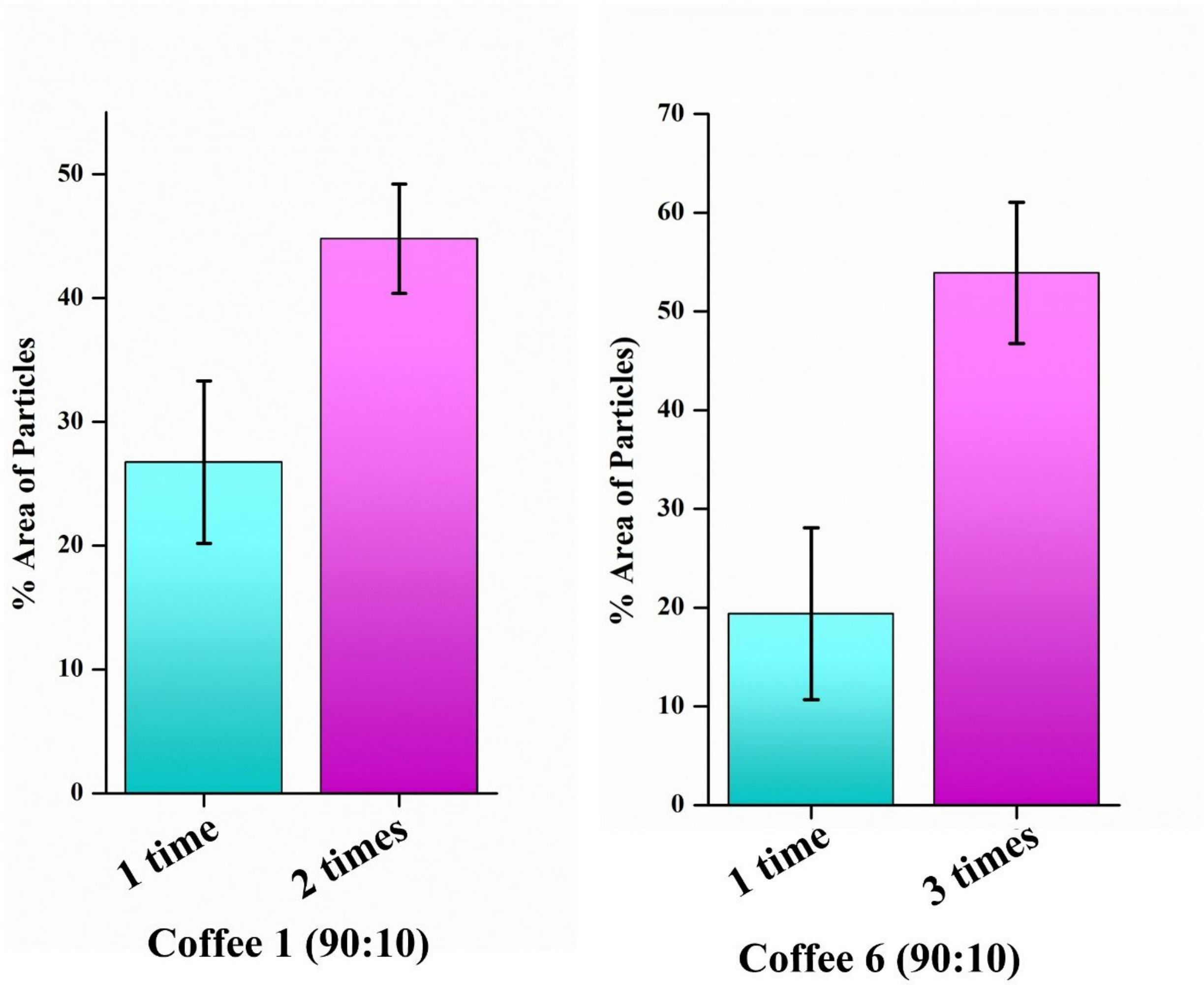

Figure S1. Percentage area adherence of **(a)** one micro spatula (~ 0.03g) and two micro spatula (~0.06g) of Coffee 1 (C1)-chicory sample **(b)** one micro spatula (~ 0.03g) and three micro spatula (~0.09g) of Coffee 6 (C6)-chicory sample.

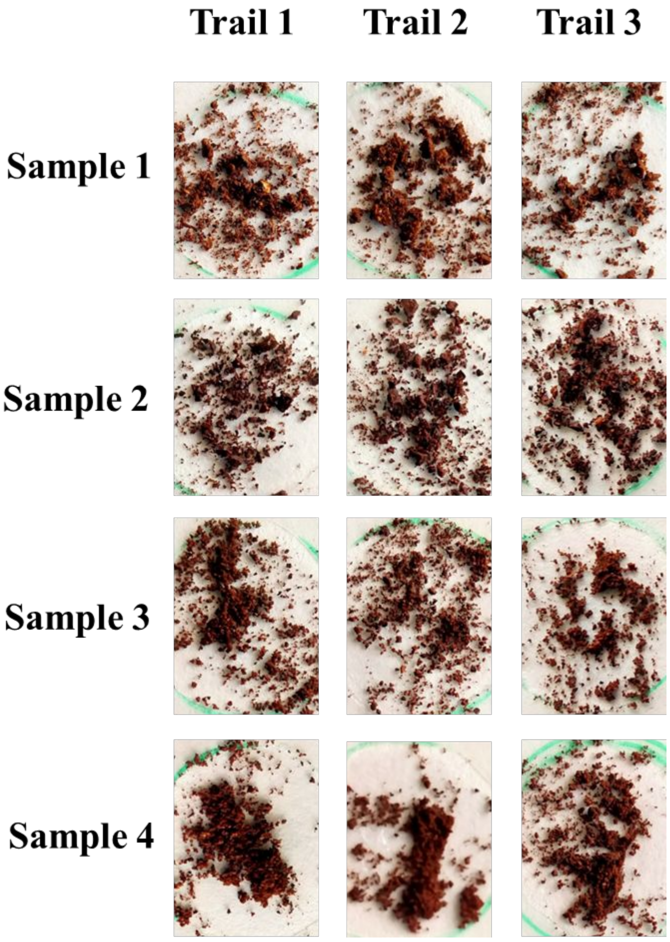

Figure S2. Testing with commercial coffee samples.
